# Association of Cerebrospinal Fluid BACE1 with Amyloid pathology and Neurodegeneration in Alzheimer disease

**DOI:** 10.1002/alz.085733

**Published:** 2025-01-09

**Authors:** Feng Gao, Yong Shen, Mengguo Zhang

**Affiliations:** ^1^ University of Science and Technology of China, Hefei China; ^2^ Neurodegenerative Disorder Research Center, Division of Biological and Medical Sciences, University of Science and Technology of China, Institute on Aging and Brain Disorders, First Affiliated Hospital of University of Science and Technology of China, Hefei China; ^3^ University of Science and Technology of China, Hefei, Anhui China

## Abstract

**Background:**

The exact impacts of β‐site APP cleaving enzyme (BACE1) on brain atrophy and cognitive decline in the Alzheimer’s disease (AD) remain not fully understood. This study aimed to investigate the relationship between BACE1 in cerebrospinal fluid (CSF) and amyloid‐β (Aβ) pathology, neurodegeneration and cognitive function.

**Method:**

This study involved 359 participants from original individuals of the China Aging and Neurodegenerative Disorder Initiative (CANDI) cohort, who underwent measurements of AD biomarkers. Among them, 259 individuals underwent baseline structural magnetic resonance imaging (MRI) scans, 101 individuals underwent ^18^F‐labeled florbetapir (AV45) positron emission tomography (PET) scans and 117 individuals received ^18^F‐labeled fluorodeoxyglucose (FDG) PET scans. Additionally, 95 participants underwent follow‐up cognitive tests, and 81 participants received follow‐up structural MRI scans.

**Results:**

Out of the 359 participants, 216 (60.17%) were women. The mean [SD] age was 62.18 [8.48] years, and the mean [SD] follow‐up times for MRI and cognitive tests were 1.52 (0.55) years and 1.79 (0.87) years, respectively. CSF BACE1 levels were elevated in patients with mild cognitive impairment caused by AD or AD. CSF BACE1 levels were correlated with soluble Aβ40 (β = 0.469, p < .001) and Aβ42 (β = 0.174, p = .001), but not AV45‐PET signal. Furthermore, CSF BACE1 levels were positively associated with cortical thickness in specific brain regions, including the insula and supramarginal areas. BACE1 was also linked to cortical glucose metabolism in frontal and supramarginal areas after adjusting regional Aβ burden. Additionally, the longitudinal study revealed that elevated BACE1 levels were associated with reduced cortical thinning in specific brain regions and less cognitive decline (β = ‐0.219, p = .05) over time.

**Conclusions:**

While increased CSF BACE1 is involved in promoting Aβ production, higher levels of BACE1 are associated with better cortical thickness and cognitive function. Maintaining the normal physiological function of BACE1 in the brain is important for cognitive function.

